# The protective role of PYY in intestinal mucosal defects induced by SATB2 deficiency in inflammatory bowel disease

**DOI:** 10.1038/s41420-025-02511-y

**Published:** 2025-05-09

**Authors:** Yao Liu, Lanqing Wu, Xiaoli Li, Yongyu Chen, Ruidong Chen, Caiyun Lv, Juan Chen, Xinjuan Fan, Guangxin Duan, Fan Zhong, Qi Sun, Qianyun Shi, Hengli Ni, Lina Sun, Jiaying Xu, Wen Tang, Jianming Li

**Affiliations:** 1 Department of Pathology, Nanjing Drum Tower Hospital, the Affiliated Hospital of Nanjing University Medical School, Nanjing, Jiangsu China; 2 Department of Pathology and Pathophysiology, Medical College of Soochow University, Suzhou, Jiangsu China; 3 Department of Gastroenterology, The Second Affiliated Hospital of Soochow University, Suzhou, Jiangsu China; 4 Department of Pathology, The Six Affiliated Hospital of Sun Yat-Sen University, Guangzhou, Guangdong China; 5 State Key Laboratory of Radiation Medicine and Protection, School of Radiation Medicine and Protection, Collaborative Innovation Center of Radiation Medicine of Jiangsu Higher Education Institutions, Soochow University, Suzhou, Jiangsu China

**Keywords:** Pharmacology, Cell biology

## Abstract

Impaired colonic mucosal repair is a critical issue in inflammatory bowel diseases (IBD). SATB2 is essential for maintaining colonic epithelial homeostasis, but its role in mucosal repair is unclear. In this study, flow cytometry was used to assess SATB2’s role in colonic epithelial repair in a radiation injury model. SATB2 knockout mice exhibited defective epithelial repair, with a marked reduction in goblet and enteroendocrine cells. Mechanistically, SATB2 directly regulated PPAR-γ transcription, and PYY was observed to translocate into the nucleus and promote the transcription of PPAR-γ target genes. In organoids derived from patients with Crohn’s disease, PYY supplementation significantly improved epithelial regeneration, outperforming the PPAR-γ agonist rosiglitazone. In conclusion, SATB2 deficiency impairs colonic epithelial repair, which can be rescued by PYY through activation of PPAR-γ-dependent transcription. These findings suggest that PYY may serve as a promising therapeutic molecule to promote epithelial repair in IBD.

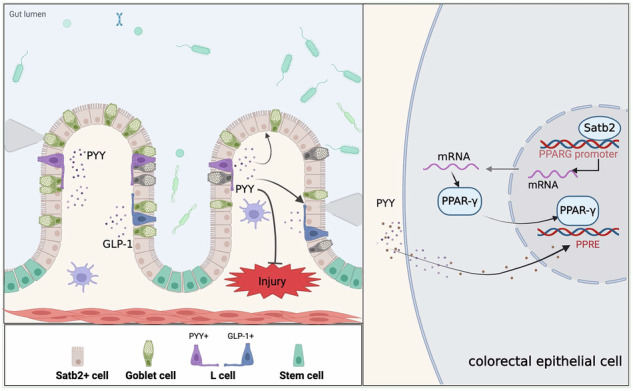

## Introduction

The integrity of the colonic mucosa is crucial for maintaining intestinal homeostasis, yet it is frequently disrupted in inflammatory bowel disease (IBD), including ulcerative colitis (UC) and Crohn’s disease (CD) [1]. Persistent mucosal injury and inadequate epithelial regeneration contribute to disease progression and complications, often necessitating surgical intervention. Despite advances in IBD management, the molecular mechanisms underlying colonic mucosal repair remain poorly understood.

One key player in maintaining colonic epithelial homeostasis is Special AT-rich sequence-binding protein 2 (SATB2), a nuclear matrix-associated transcriptional regulatory factor. SATB2 interacts with nuclear matrix attachment regions in the mammalian genome, which aids in shaping of chromatin structure and recruiting chromatin remodeling factors to control gene expression [2]. Under physiological conditions, SATB2 is widely expressed in colonic epithelial cells, while it is absent in small intestinal epithelial cells. A recent study has demonstrated that the maintenance of tissue specificity in colonic epithelium during renewal relies on the expression of SATB2. The loss of SATB2 leads to features reminiscent of the small intestinal mucosa in the colonic mucosa [3]. Our previous research found that the deficiency of colonic SATB2 disrupts the expression of chloride ion exchangers and promotes the colonization of bacteroids in the intestine, thereby promoting the onset and progression of ulcerative colitis [4]. Despite these insights, the specific roles of SATB2 and its regulated molecules in the repair of the colonic mucosa remains unclear, necessitating further research.

During the repair of the colonic mucosa, Lgr5-positive intestinal stem cells are essential [5]. Colonic epithelial cells continuously migrate to the surface and differentiate into various functional cells, including absorptive cells, goblet cells, enteroendocrine cells, and tuft cells [6]. Enteroendocrine cells, characterized by their low numbers and indistinct morphology, have been less studied. Various types of enteritis may also lead to damage to enteroendocrine cells, resulting in disturbances in intestinal function. Recent studies have revealed the regulatory role of enteroendocrine cells in other types of intestinal epithelial cells, like tuft cells [7]. Currently, the specific functions of enteroendocrine hormones in the repair of the colonic mucosa remain poorly understood, warranting further investigation.

## Results

### The expression level of Satb2 was correlated with the degree of colonic mucosal repair after irradiation injury

After exposure to 8 Gy irradiation, C57BL/6 mice were sacrificed at 2, 3, 5, and 7 days post-irradiation (dpi). The baseline proliferative index of colonic epithelial cells was initially around 50%, but it decreased to approximately 20% on 2–3 dpi, then increased to about 70% on 5 dpi, and returned to normal levels (around 55%) on 7 dpi (Supplementary Fig. 1A, B). HE staining showed necrosis and shedding of colonic epithelial cells, along with atrophic changes in the intestinal mucosa on day 3, indicative of the damage phase. By day 5, the colonic epithelial cells showed enlarged nuclei, increased cell density, and hyperplastic changes in the intestinal mucosa, representing the repair phase (Supplementary Fig. 1C, D). This mice model exhibited a temporal sequence of colonic mucosal injury and repair.

**Fig. 1:**
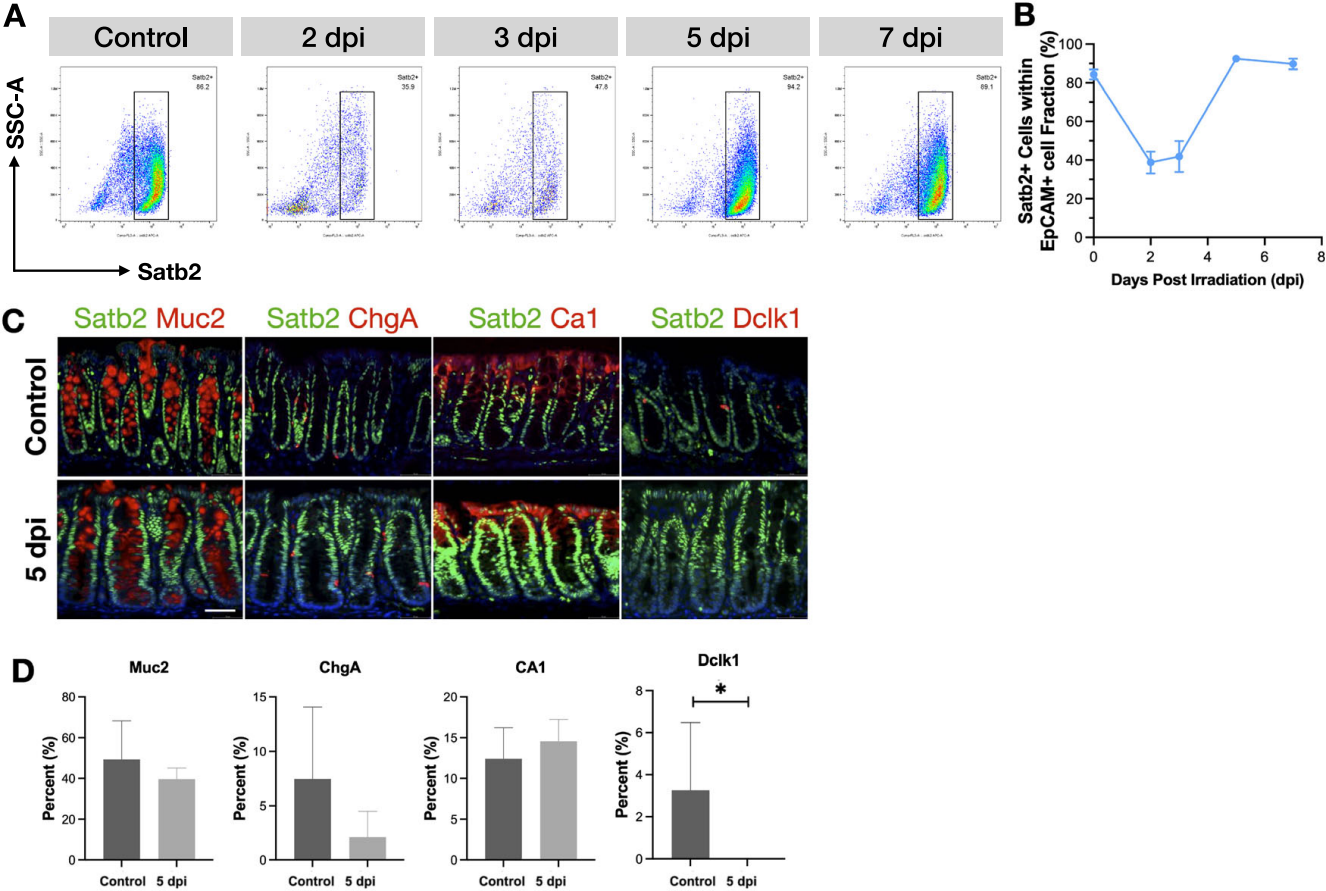
Analysis of Satb2 expression and cell types in the colonic mucosa following irradiation. **A** Flow cytometry analysis of Satb2-positive cells within EpCAM-positive cells from the colon of C57BL/6 mice at different days post-irradiation (dpi). **B** Proportion of Satb2-positive cells at the indicated dpi. *n* = 5 mice per dpi. one-way ANOVA, *P < 0.05. **C** Representative images of co-staining for Satb2 and Muc2 (goblet cells), Ca1 (colonocytes), Chga (enteroendocrine cells), or Dclk1 (tuft cells) in the distal colon of C57BL/6 mice before irradiation and at 5 dpi. **D** Quantification of Muc2, ChgA, CA1, or Dclk1-positive cells before irradiation and at 5 dpi. *n* = 10 crypts per group, *t*-test, *P < 0.05.

Flow cytometry analysis revealed a dynamic expression pattern of Satb2 in colonic epithelial cells following irradiation. On 2–3 dpi, Satb2 expression declined from approximately 85% in the physiological state to about 40%, reaching around 95% by 5 dpi, and returning to baseline levels (approximately 90%) by 7 dpi (Fig. 1A, B). This temporal regulation of Satb2 expression closely correlated with the rectal mucosa’s damage and repair phases. Notably, during the repair phase, although the tuft cell marker Dclk1 remained suppressed, other markers, including Muc2 for goblet cells, Ca1 for absorptive cells, and ChgA for enteroendocrine cells, were expressed and co-localized with Satb2 (Fig. 1C, D). This implies that Satb2-positive cells, including goblet cells, absorptive cells, and enteroendocrine cells, might be involved in the regeneration of the colonic mucosa.

### Satb2 deficiency resulted in impaired repair of the colonic mucosa after irradiation

**Fig. 2:**
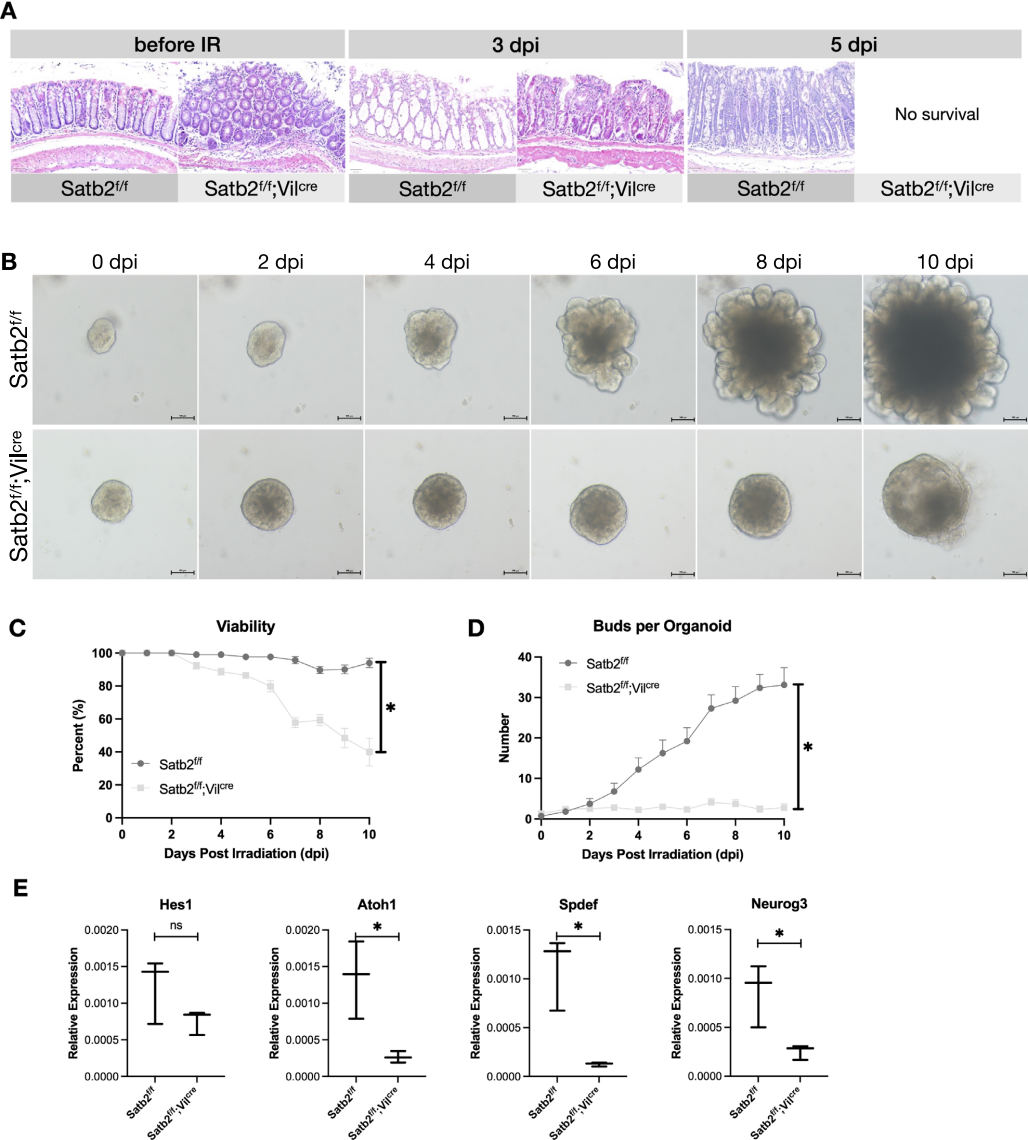
Effects of Satb2 deletion on colonic mucosal repair following ionizing radiation. **A** H&E staining of colonic tissues from Satb2^f/f^ and Satb2^f/f^;Vil^cre^ mice at baseline, 3 days, and 5 days post-irradiation. **B** Growth of colonic organoids in Satb2 knockout and control mice after irradiation. **C** Statistical analysis of crypt survival rates between the two groups. *n* = 10 fields of view per group, one-way ANOVA, *P < 0.05. **D** Number of organoid buds in both groups at day 10 post-irradiation. *n* = 10 organoids per group, one-way ANOVA, *P < 0.05. **E** Quantitative PCR analysis of intestinal epithelial lineage markers (Hes1 for absorptive lineage, Atoh1 for secretory lineage, Spdef for goblet cells, and Neurog3 for enteroendocrine cells) in organoids collected on day 10 post-irradiation. *n* = 3 replicates per group, *t*-test, *P < 0.05.

As shown in our previous study [4], Satb2^f/f^;Vil^Cre^ mice exhibited inflammation in the colonic mucosa before irradiation. On 3 dpi, colonic epithelial cells displayed evident eosinophilic alterations, accompanied by significant inflammatory cell infiltration in the lamina propria. All Satb2^f/f^;Vil^Cre^ mice mice died on day 4 post-irradiation (Fig. 2A). We further conducted in vitro experiments to exclude potential confounding factors from in vivo experiments. Colonic crypts were isolated from Satb2^f/f^ and Satb2^f/f^;Vil^Cre^ mice for organoid culture. After 2 Gy radiation injury, the Satb2 KO colonic organoids exhibited markedly smaller volumes with few buddings (Fig. 2B). Both the survival rate and budding numbers of organoids significantly decreased (Fig. 2C, D). RNA collected from the organoid cultures on 10 dpi revealed reduced expression levels of secretory lineage markers, including Spdef for goblet cells and Neurog3 for enteroendocrine cells, suggesting a significant reduction in secretory lineage cells following Satb2 deficiency (Fig. 2E). Therefore, Satb2 knockout may limit the number of secretory lineage cells in colonic mucosa, thereby impeding mucosal repair.

### The colonic endocrine hormone PYY decreased following Satb2 deletion or during colonic mucosal injury

**Fig. 3:**
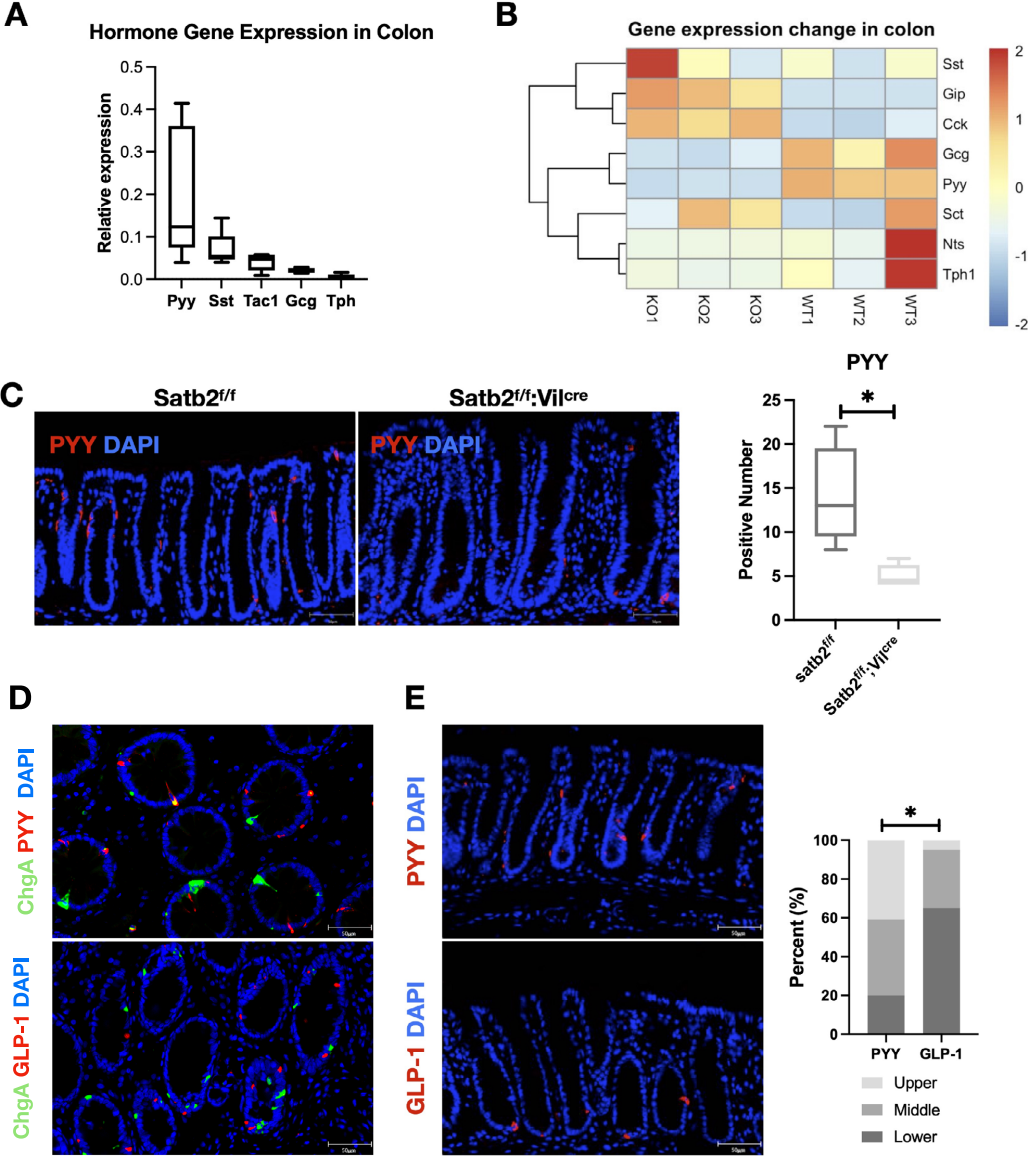
Changes in colonic endocrine hormones following Satb2 knockout. **A** qPCR analysis of endocrine hormone expression in the colonic mucosa of C57BL/6 mice. *n* = 3 mice per group. **B** Heatmap depicting the changes in the expression of colonic endocrine hormones following Satb2 knockout, derived from our RNA-seq data. **C** Immunofluorescence analysis showing the number of PYY-positive cells in Satb2 IEC-KO and control mice, along with statistical analysis. *n* = 20 fields of view per group, *t*-test, *P < 0.05. **D** Immunofluorescence colocalization of L-cell-secreted hormones and the classic neuroendocrine marker ChgA. **E** Immunofluorescence images showing the localization of PYY and GLP-1 in the colonic mucosa (left), and the corresponding statistical analysis (right). *n* = 20 fields of view per group, Chi-square test, *P < 0.05.

The colonic mucosa harbors diverse enteroendocrine cells with indistinct morphologies, including enterochromaffin cells (producing 5-HT), D cells (producing SST), and L cells (producing GLP-1 and PYY). Among these, PYY is the most abundant hormone, as evidenced by evaluating enteroendocrine hormone expression in C57BL/6 mouse colonic mucosa (Fig. 3A). After Satb2 knockout, both the expression levels of PYY and GLP-1 secreted by L cells significantly decreased (Fig. 3B). Immunofluorescence analysis confirmed a significant reduction in PYY-positive cells in the colonic mucosa after Satb2 knockout (Fig. 3C). Traditional pathological enteroendocrine marker ChgA failed to fully identify PYY and GLP-1 (Fig. 3D). Despite both hormones being secreted by L cells, they exhibited distinct colonic mucosa localization, with PYY primarily in the superficial layer and GLP-1 tending to be located in the basal layer (Fig. 3E). Given that colonic mucosal damage typically initiates at the crypt surface and progresses to the base in response to harmful factors, PYY-positive cells in the superficial layer may be more susceptible to loss compared to GLP-1-positive cells in the basal layer.

We also observed a reduction in the number of PYY-positive cells during various colon mucosal injuries. In mouse colonic mucosa, PYY-positive cell numbers significantly decreased during the injury phase of radiation-induced colitis but recovered during the repair phase (Supplementary Fig. 2A). Utilizing IL10-deficient mice, a classic model for ulcerative colitis, we found a substantial reduction in the number of PYY-positive cells in colonic tissues compared to control mice (Supplementary Fig. 2B). Analysis of specimens from chronic radiation-induced colitis and control colonoscopy biopsies showed that PYY expression, predominantly located in the upper part of normal crypts, diminished significantly in chronic radiation-induced colitis, correlating with the severity of colonic mucosal damage (Supplementary Fig. 2C).

### Satb2 deletion decreased the energy metabolism of colonic epithelial cells by down-regulating the transcription of PPARG

In further exploring how Satb2 contributes to intestinal mucosal repair, we initially hypothesized that its increased expression might promote the proliferation of colonic epithelial cells during the repair process following irradiation. However, Ki67 and BrdU staining on Satb2 knockout and control colonic tissues revealed no significant differences in proliferation indices (Supplementary Fig. 3A–D). Additionally, overexpressing Satb2 had minimal impact on cell proliferation assessed by CCK8 (Supplementary Fig. 3E, F). These results suggest that Satb2 does not significantly affect colonic epithelial cell proliferation during mucosal repair.

**Fig. 4:**
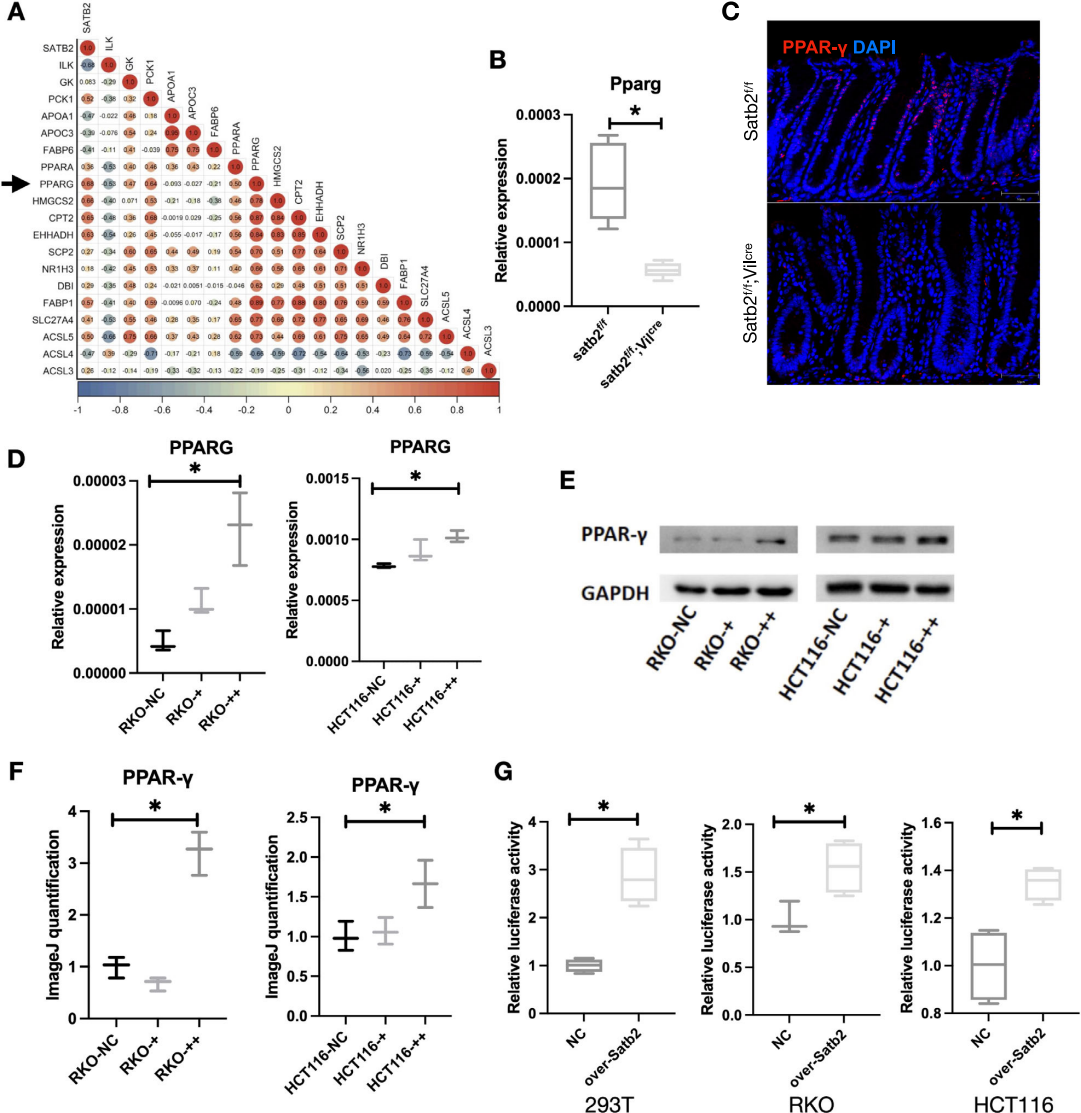
The role and mechanism of Satb2 regulation of PPARG expression. **A** Correlation analysis between Satb2 expression and metabolism-related genes based on TCGA data from 43 adjacent colorectal cancer tissues. **B** qPCR analysis of Pparg expression levels in colonic mucosal tissues from Satb2^f/f^ and Satb2^f/f^;Vil^cre^ mice. *n* = 3 mice per group, *t*-test, *P < 0.05. **C** Immunofluorescence detection of PPAR-γ expression in colonic mucosal tissues from Satb2^f/f^ and Satb2^f/f^;Vil^cre^ mice. **D** qPCR analysis of PPARG RNA expression in RKO and HCT116 cells after overexpression of Satb2. *n* = 3 replicates per group, one-way ANOVA, *P < 0.05. **E** Western blot analysis of PPAR-γ protein expression levels in RKO and HCT116 cells following Satb2 overexpression. **F** Statistical analysis of western blot band intensity results. *n* = 3 replicates per group, one-way ANOVA, *P < 0.05. **G** Luciferase reporter assay of PPARG activity in 293 T, RKO, and HCT116 cells following Satb2 overexpression. *n* = 4 replicates per group, *t*-test, *P < 0.05.

Our RNA-seq analysis uncovered the impact of Satb2 knockout on the expression of numerous genes in colonic tissues. Considering the vital role of energy requirement in the differentiation of intestinal epithelium, we extracted the differentially expressed metabolism-related genes and further examined their correlation with Satb2 expression in RNA-seq data from 43 adjacent colorectal cancer tissues in the Cancer Genome Atlas (TCGA). The results revealed a significant positive correlation between Satb2 and the key energy metabolism transcription factor, PPARG (Fig. 4A). Subsequent quantitative PCR demonstrated a noteworthy decrease in Pparg expression following Satb2 knockout in mouse colonic mucosal tissues (Fig. 4B). Additionally, there was a conspicuous reduction in PPAR-γ-positive cells in the colonic mucosa after Satb2 knockout (Fig. 4C). In RKO and HCT116 cell lines, PPARG expression increased with Satb2 upregulation (Fig. 4D). Immunoblotting indicated that Satb2 overexpression led to increased PPAR-γ protein levels (Fig. 4E, F). Luciferase reporter gene plasmid experiments confirmed that Satb2 significantly increased the luciferase activity of the PPARG promoter, suggesting Satb2’s role in promoting PPARG transcription (Fig. 4G).

### PYY demonstrated nuclear entry and led to the upregulation of downstream genes of PPAR-γ

**Fig. 5:**
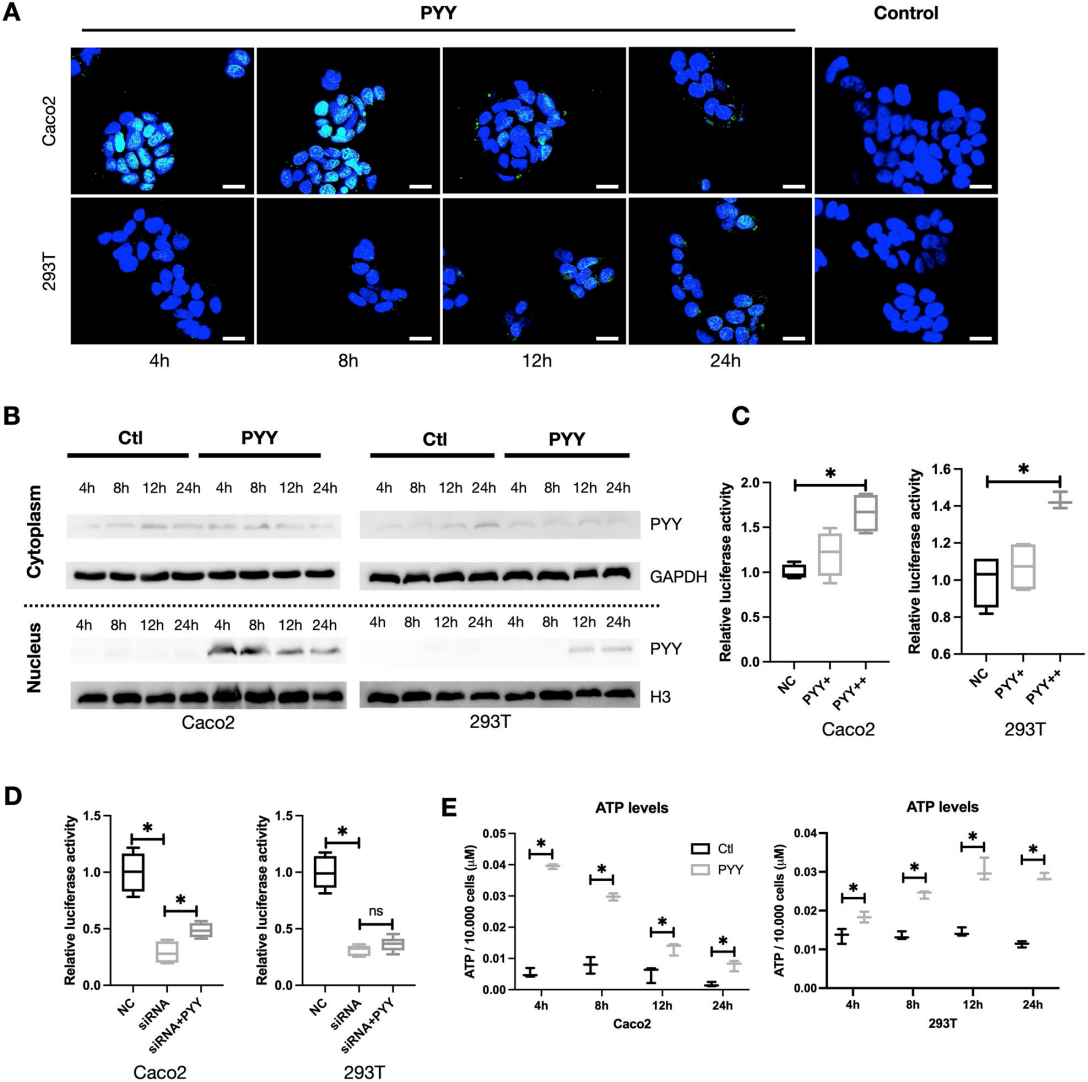
The transcriptional mechanism of PYY regulation of PPAR-γ target genes. **A** Confocal microscopy images showing PYY nuclear translocation at different time points following PYY supplementation in cell culture medium. **B** Nuclear-cytoplasmic fractionation and Western blot analysis of PYY expression in Caco2 and 293 T cells after PYY treatment. **C** Luciferase reporter assay measuring PPAR activity in Caco2 and 293T cells after PYY supplementation. *n* = 6 replicates per group, one-way ANOVA, *P < 0.05. **D** Luciferase reporter assay measuring PPAR activity in Caco2 and 293T cells with Satb2 knockdown and PYY treatment. *n* = 6 replicates per group, one-way ANOVA, *P < 0.05. **E** Measurement of ATP levels in Caco2 and 293T cells treated with PYY. *n* = 6 replicates per group, t-test, *P < 0.05.

As an endocrine hormone, it is currently unclear whether PYY can improve the metabolism of colonic epithelial cells. Therefore, we designed the following experiment. First, we modified the N-terminus of PYY short peptide with FITC-Ahx. Subsequent confocal imaging showed the intracellular translocation of PYY into the nucleus (Fig. 5A). This finding was confirmed by nuclear-cytoplasmic fractionation experiments, emphasizing an enriched presence of PYY in the nucleus (Fig. 5B). The timeframe for PYY nuclear entry exhibited variability across distinct cell lines, with penetration into the nucleus occurring within 4 h in Caco2 cells and requiring a minimum of 12 h in 293T cells (Fig. 5A, B)

To explore the PYY-PPRE relationship, we used a luciferase reporter gene (pGMPPAR) and added PYY during the luciferase assay. The results showed a concentration-dependent increase in PPRE activity with higher PYY concentrations (Fig. 5C), indicating PYY’s partial role as a PPRE activator. After interfering Satb2 expression, 293T and Caco2 cells were also treated with 100 pmol of PYY, and PPRE’s luciferase activity was assessed. Satb2 interference reduced PPRE activity, and adding PYY post Satb2 knockdown increased PPRE luciferase activity. However, achieving complete restoration to pre-Satb2 knockdown levels was challenging (Fig. 5D). These results suggest that PYY can directly activate PPRE in colonic epithelium, as evidenced by the upregulation of PPRE downstream genes and the increased ATP production following PYY treatment (Fig. 5E).

### PYY promoted the repair of Satb2 KO colonic organoids after irradiation and mitigates DSS-induced colonic mucosal damage

**Fig. 6:**
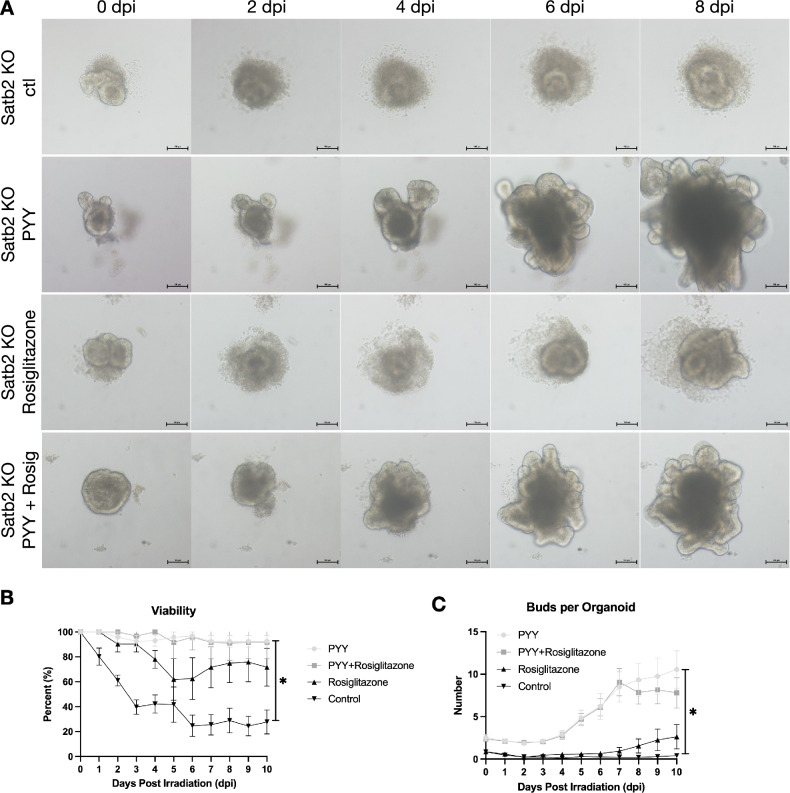
Effects of PYY treatment on the repair of Satb2-deficient colonic organoids after ionizing radiation. **A** Organoids were cultured from colon crypts of Satb2^f/f^;Vil^cre^ mice and treated with PYY, a PPAR-γ agonist, or both, followed by a single 2 Gy dose of irradiation. Organoid images were taken at different time points post-irradiation. **B** Statistical analysis of organoid survival rates. *n* = 20 fields of view per group, one-way ANOVA, *P < 0.05. **C** Statistical analysis of organoid budding counts. *n* = 20 organoids per group, one-way ANOVA, *P < 0.05.

Molecular experiments confirmed that PYY can rescue the metabolic dysfunction caused by Satb2 deficiency in colonic epithelium, but its role in epithelial repair remains unclear. We further investigated this through in vitro and animal experiments. We isolated intestinal crypts from Satb2^f/f^;Vil^cre^ mice for organoid culture and added PYY, PPAR-γ agonist (rosiglitazone), or a combination of PYY and rosiglitazone to the organoid culture medium. Colonic organoid morphology during the repair phase after radiation exposure was observed (Fig. 6A). Before irradiation (0 dpi), organoid morphology was similar across groups. PYY significantly improved organoid survival and budding post-irradiation, surpassing the effects of rosiglitazone (Fig. 6B, C). This suggests that PYY could effectively ameliorate the intestinal epithelial repair defect caused by Satb2 deficiency, outperforming the PPAR-γ agonist rosiglitazone. Hence, PYY emerged as a potential strategy for promoting colonic mucosal repair.

Subsequently, in the DSS-induced colitis model, we found that intraperitoneal injection of PYY alleviates weight loss and results in a longer colon length (Supplementary Fig. 4A, B). Microscopic examination of mouse colonic tissue reveals reduced inflammatory cell infiltration, maintained crypt morphology, and fewer surface defects in the PYY group compared to controls (Supplementary Fig. 4C, D). The crypt damage scoring indicates a significant reduction in whole crypt damage in the PYY group (38% vs. 74% in the control group). Regarding the depth of inflammatory infiltration, in the control group, 84% of crypts have inflammation infiltrating to the muscular layer, even transmurally, while in the PYY group, only 30% of crypts reached this depth, 56% extend to the submucosal layer, and 14% are limited to the mucosal layer. Overall, the inflammation score is lower in the PYY group (Supplementary Fig. 4D). The expression levels of inflammatory mediators, including IL-1β, Il-6 and TNF-α, in intestinal tissues are also lower (Supplementary Fig. 4E). Therefore, PYY could significantly alleviate colonic mucosal damage and promote repair.

### PYY can improve intrinsic defects in the intestinal epithelium of clinical colitis specimens

**Fig. 7:**
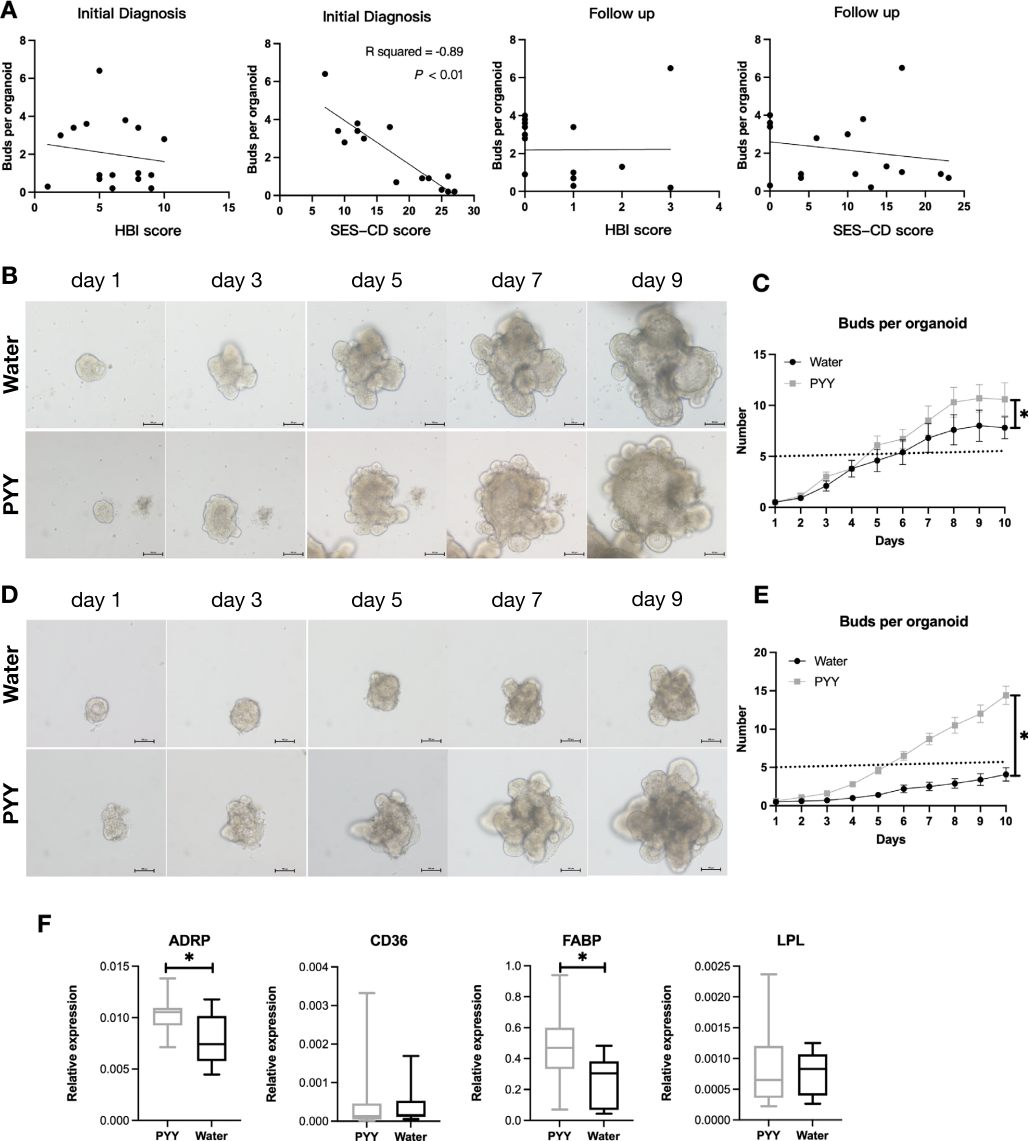
Effects of PYY treatment on the activity of organoids derived from human Crohn’s disease colon biopsy specimens. **A** Correlation analysis between the organoid budding rate and clinical symptom scores (*n* = 16), endoscopic scores (*n* = 14) at the initial diagnosis, and clinical (*n* = 17) and endoscopic (*n* = 17) scores at the current examination. **B**, **C** Morphological changes of high-activity organoids after passage and treatment with PYY or control (**B**). Statistical analysis of organoid budding rates following PYY treatment. *n* = 10 organoids per sample (**C**). **D**, **E** Morphological changes of low-activity organoids after passage and treatment with PYY or control (**D**). Statistical analysis of organoid budding rates following PYY treatment. *n* = 10 organoids per sample (**E**). **F** Quantitative PCR analysis of the expression levels of PPAR-γ downstream genes in organoid RNA extracts after PYY treatment. *n* = 5 per group, *t*-test, *P < 0.05.

Organoid cultures were derived from nineteen colon mucosal biopsies of CD patients undergoing routine follow-up, with a few being newly diagnosed patients (Supplementary Table 1). Interestingly, although most of the organoids were derived from biopsies taken during routine follow-up, the organoid budding rate showed a significant negative correlation with the endoscopic scores at the time of initial diagnosis, but no significant correlation with the clinical symptom scores or endoscopic scores during follow-up, nor with the clinical symptom scores at the time of initial diagnosis (Fig. 7A). After passaging, the organoids were divided into two groups: one group received PYY treatment, while the other served as a control. Without any treatment, the proliferative activity of the organoids varied, with some showing higher activity (Fig. 7B, top), while others displayed weaker activity (Fig. 7D, top). PYY treatment significantly enhanced the proliferative activity of organoids in both groups (Fig. 7B–E).

RNA from PYY-treated and untreated organoids underwent qPCR to assess PPAR-γ target gene expression, including ADRP, CD36, FABP, and LPL. Results revealed an increasing trend in the expression levels of these genes after PYY treatment, with statistically significant upregulation observed in ADRP and FABP (Fig. 7F). These findings suggest that PYY effectively promotes the regeneration of colonic mucosa through the PPAR-γ pathway.

## Discussion

This study found that the expression level of Satb2 was closely associated with the degree of colonic mucosal repair following irradiation injury. Satb2 deficiency impaired the repair process, which was accompanied by a reduction in the colonic endocrine hormone PYY. Moreover, the absence of Satb2 downregulated the transcription of PPARG, leading to impaired energy metabolism in colonic epithelial cells. However, supplementation with PYY significantly promoted the repair of Satb2-deficient colonic organoids after irradiation and alleviated DSS-induced colonic mucosal damage. PYY was also shown to enter the nucleus and upregulate the downstream genes of PPAR-γ, suggesting its role in enhancing epithelial repair. Additionally, PYY improved intrinsic defects in the intestinal epithelium of clinical colitis specimens, further supporting its potential as a therapeutic strategy for colonic mucosal repair.

During colonic mucosal repair, Lgr5-positive intestinal stem cells, driven by Wnt signaling, play a crucial role [8]. Activation of the Notch pathway is also indispensable during this phase of tissue repair, as evidenced by increased expression of Notch ligands and the activated form of Notch1 in the inflamed colonic mucosa. Blocking global Notch activation resulted in a severe loss of the proliferative response in the colitic mucosa, exacerbating DSS colitis [9]. Additionally, BMP signaling may be involved in colonic epithelial repair, as indicated by the improved colonic organoid formation when treated with BMP antagonists secreted by mesenchymal stromal cells [10]. Although Satb2 is considered a downstream effector of BMP signaling [11], its specific role in intestinal mucosal repair requires meticulous exploration. Future studies should further explore the downstream regulatory networks of Satb2 to elucidate its broader role in colonic mucosal repair.

Unlike classical proliferation-related repair mechanisms, our findings suggest that Satb2 primarily influences the differentiation of enteroendocrine cells, rather than directly modulating epithelial proliferation. The RNA-seq data from Satb2 knockout mice indicated significant alterations in the transcriptional levels of enteroendocrine hormones. Despite constituting only around 1% of all intestinal epithelial cells, enteroendocrine cells play a crucial role in the body’s metabolism through the hormones they secrete. Subsequently, our investigation delved into the role of PYY in colonic mucosal injury repair. Intact PYY, a 36-amino acid peptide belonging to the neuropeptide Y family, exists in two forms in vivo: PYY1-36 and PYY3-36. Recent studies have revealed that PYY1-36 can be produced by the Paneth cells of the small intestine, exhibiting antifungal effects [12]. Traditionally, it was believed that after a meal, L cells release PYY3-36 into the cryptal basolateral side containing capillaries, entering the systemic circulation and exerting appetite-suppressing effects [13]. Our findings suggest that PYY may function similarly to PPAR-γ, activating DNA-specific PPAR-γ response elements.

PPARs (PPAR-γ, PPAR-α, and PPAR-δ) are ligand-dependent nuclear transcription factors that regulate gene expression in various processes, including lipid and glucose metabolism, atherosclerotic plaque formation, inflammation, cytokine differentiation, and more [14]. PPAR-γ has diverse roles, including adipocyte differentiation, expression of mitochondrial uncoupling proteins, downregulation of leptin, and interaction with multiple genes controlling insulin sensitivity [14]. Local administration of the PPAR-γ agonist pioglitazone has been shown to reduce epidermal proliferation and decrease T lymphocyte-mediated inflammation [15].

Although elevated levels of peripheral blood PYY have been reported in Crohn’s disease patients early on [16], the number of PYY-positive cells in intestinal tissues shows a declining trend [17]. Hence, the levels of PYY in peripheral blood and the number of PYY-positive cells in intestinal mucosal tissues may not be consistent. Recent studies suggest that GLP-1-based therapy might improve the disease progression of IBD [18]. Beyond its anti-inflammatory effects, PYY may exhibit anti-cancer properties. Research indicates that exogenous PYY can inhibit the proliferation and metastasis of colorectal cancer cells, enhancing cancer cells apoptosis [19]. PYY treatment also demonstrates inhibitory effects on the proliferation of breast cancer [20]. These research findings broaden our understanding of intestinal endocrine hormones.

In conclusion, our study establishes the protective role of Satb2 in intestinal mucosal repair by regulating PPARG. Satb2 deficiency reduces PYY levels, and PYY supplementation promotes mucosal repair by activating PPARE. These findings suggest potential therapeutic strategies for chronic colonic injuries.

## Methods

### Mice models

All mice were housed in the barrier facilities at the Laboratory Animal Center in Soochow University. Due to the exploratory nature of this study, a formal sample size calculation was not performed, and the sample size was chosen based on feasibility and prior studies [4]. We used a randomization strategy by a random number generator to allocate mice to treatment and control groups to minimize potential bias in the assignment process. The order of treatments was also randomized to avoid any systematic effects due to treatment sequence. The intestinal epithelium-specific Satb2 knockout mice (Satb2^f/f^;Vil^Cre^) used in this study have been previously described in our research [4]. The IL10 knockout mice (IL10^-/-^) employed in this study belong to the classic model of ulcerative colitis.

The radioactive intestinal injury model was established as described in previous publications [21]. In brief, male C57BL/6 mice aged ten to twelve weeks were restrained in custom-made plastic mouse holders and placed inside a biological X-ray irradiator (Rad source, RS-2000 Pro). The irradiation parameters included a source-to-skin distance of 40 cm, an absorbed dose rate of 200 cGy/min, a total body irradiation duration of 392 s, and a single cumulative radiation dose of 8 Gy.

The chemical-induced intestinal injury model was induced by dextran sulfate sodium (DSS) (MP, USA), as in our previous publications [4, 22]. In the DSS-induced colitis model, intraperitoneal injections of PYY were administered, with each mouse receiving 100 µL of 0.1 nmol PYY per 20 grams of body weight. The intestinal hormone PYY3-36 powder was obtained from Suzhou Zhongchu Zhiyuan Technology Co., Ltd, and it has the following sequence (N’- IKP EAP GED ASP EEL NRY YAS LRH YLN LVT RQR Y - C’).

### Patients and specimens

Intestinal biopsy specimens were obtained from the ascending colon of 19 CD patients at the Second Affiliated Hospital of Soochow University from November 2023 to January 2024. All recruited CD patients were clinically diagnosed, and all slides were reviewed and confirmed as CD by pathologists specializing in IBD. Basic information and clinical/endoscopic scores at initial diagnosis and follow-up in Crohn’s disease patients was shown in Supplementary Table 1. The Simplified Crohn’s Disease Activity Index (Harvey-Bradshaw Index, HBI) and the Simple Endoscopic Score for Crohn’s Disease (SES-CD) were recorded for Crohn’s disease patients. Biopsy specimens were obtained from the ascending colon and were all in the remission phase of CD. Formalin-fixed paraffin-embedded (FFPE) surgical specimens of radiation enteritis were obtained from the Department of Pathology at the Sixth Affiliated Hospital of Sun Yat-Sen University. Patients or the public were not directly involved in the design, conduct, or dissemination of this study.

### Ethics approval and consent to participate

All methods were performed in accordance with relevant guidelines and regulations. Separate ethics approvals were obtained for animal and human studies from their respective institutional committees. Ethical approval for all animal experiments was obtained from the Committee on Animal Research of Soochow University. Human sample collection and related experiments were approved by the Ethics Committee of the Second Affiliated Hospital of Soochow University (Ethics approval number: JD-HG-2024-013). Informed consent was obtained from all participants for the use of their samples in this research.

### Plasmids

The cDNA of SATB2 was kindly provided by the laboratory of Prof. Jiahuai Han (Xiamen University, China). The promoter of PPARG was synthesized and cloned into pGL6-luc vectors. The PPAR-Luc construct, designed for assessing PPAR transcriptional activity, incorporates multiple PPAR binding sites, also known as DNA-specific PPAR response elements (PPRE), inserted at its multicloning sites. This construct was obtained from Yeasen Biotech. After transfecting this plasmid into the cell lines and simultaneously adding PYY, this experiment is conducted to investigate whether PYY binds to the PPRE.

### Confocal

PYY3-36 is the active form of PYY, and we synthesized its sequence with FITC-Ahx modification at the N-terminus. Subsequently, the FITC-Ahx-modified PYY was added to the culture medium of Caco2 and 293T cell lines. After treating the cells for 4, 8, 12, and 24 h, they were washed with PBS and other buffer solutions to remove unabsorbed markers. Following fixation with 4% formaldehyde at room temperature for 15 min and subsequent PBS washing, DAPI was applied for staining at room temperature for 10 min. After further buffer solution washing, slides were sealed in the dark throughout the process. Samples were then placed on a laser confocal microscope, excited with a 488 nm wavelength, and fluorescence signals were sequentially captured in optical slices to obtain spatial distribution within the cells and nuclei.

### Bioinformatics and statistical analysis

Differentially expressed genes were identified using DESeq2. Genes related to metabolism were sourced from the KEGG database. The colorectal cancer RNA-seq data utilized in this study were obtained from TCGA with the version dated 20160128. Gene co-expression analysis was conducted by calculating the Pearson correlation coefficient, and the resulting correlation heatmap was generated using the R corr package. All statistical analyses were performed using R software (version 4.3.1; the R Foundation for Statistical Computing), and a two-sided test with *P* < 0.05 was considered statistically significant.

### Summary

#### What is already known on this topic?

- Mucosal regeneration is impaired in chronic colitis, and the loss of Satb2 significantly inhibits epithelial repair.
- Existing treatments for chronic colitis are limited, and improving epithelial regeneration remains a key challenge.

#### What this study adds?

- This study reveals that Satb2 deficiency leads to reduced production of PYY, an enteroendocrine hormone.
- PYY enhances the activity of human colonic organoids derived from endoscopic biopsy specimens.
- It promotes epithelial repair in chronic colitis by entering the cell nucleus and upregulating PPARγ, which facilitates colonic epithelial regeneration.

#### How this study might affect research, practice or policy?

- PYY may offer a novel therapeutic strategy for treating chronic colitis.
- These findings could guide future research on epithelial regeneration and inform clinical approaches to managing chronic inflammatory bowel diseases (IBD).

## Supplementary information

Supplementary Materials

Supplementary Figures

Original Data

## Data Availability

All data supporting the findings of this study are available within the article and its supplementary materials. RNA-seq data used in this study have been previously published and are available in our earlier publication [4]. Additional raw data, including uncropped western blot images, are provided in the supplementary file titled “Original Data.” Further datasets are available from the corresponding author upon request. Additional methods were provided in the Supplementary Methods.
