# Supplementary material for: The protective role of PYY in intestinal mucosal defects induced by SATB2 deficiency in inflammatory bowel disease
Source: Cell Death Discov. 2025 May 9;11:227. doi: 10.1038/s41420-025-02511-y (PMC12062304; doi:10.1038/s41420-025-02511-y)
Supplement: Supplementary file 1 — Supplementary Materials [file 41420_2025_2511_MOESM1_ESM.doc]

**Supplementary Materials**

**The Protective Role of PYY in Intestinal Mucosal Defects Induced by SATB2 Deficiency in Inflammatory Bowel Disease**

Yao Liu^1,2^*†, Lanqing Wu^1,2^*, Xiaoli Li^2^*, Yongyu Chen^2^, Ruidong Chen^3^, Caiyun Lv^3^, Juan Chen^2^, Xinjuan Fan^4^, Guangxin Duan^5^, Fan Zhong^2^, Qi Sun^1^, Qianyun Shi^1^, Hengli Ni^2^, Lina Sun^2^, Jiaying Xu^5^, Wen Tang^3^†, Jianming Li^2^†

**Supplementary Figure Legend**

**Supplementary Figure 1. Histological changes in colorectal mucosa following radiation-induced injury.**

(A-B) Flow cytometric analysis of Ki67-positive cells in colorectal epithelial cells (EpCAM-positive) of C57BL/6 mice at indicated pre-and post-irradiation time points (A), with the corresponding proportion data presented in (B). n = 5 mice per dpi.

(C) HE staining (top) and Ki67 IHC detection (bottom) of colorectal tissues in C57BL/6 mice at indicated pre-and post-irradiation time points.

(D) Bar chart showing the proportion of atrophic and hyperplastic crypts in colorectal mucosa at indicated pre-and post-irradiation time points. n = 3 mice per group, t-test, **P* < 0.05.

**Supplementary Figure 2. PYY expression analysis during intestinal mucosal injury.**

(A) IF detection of PYY-positive cells during the repair process of radiation-induced injury. Red arrows indicate PYY-positive cells. n = 20 fields of view per group, ANOVA, **P* < 0.05.

(B) Quantification of Pyy IHC positive cells in the colon of C57BL/6 and IL-10 knockout mice. n = 4 mice per group, t test, **P* < 0.05.

(C) IF detection of PYY-positive cells in tissues from patients with chronic radiation colitis and control tissues from colonoscopy biopsies. Red arrows indicate PYY-positive cells, and red dashed lines indicate evident crypt damage.

**Supplementary Figure 3. Analysis of the relationship between Satb2 and the proliferative capacity of colorectal epithelial cells.**

(A-D) IF detection of Satb2 and Ki67 (A) or BrdU (C) in the colonic tissues of Satb2^f/f^;Lgr5^GFP-CreER^ mice, analyzing the proliferation rate of colorectal epithelial cells in crypts after Satb2 knockout and in control mice (B and D). n = 25 fields of view per group, t-test, **P* < 0.05.

(E-F) Overexpression of Satb2 in HCT116 cell line (E), and CCK8 assay showing the proliferation rates of the two cell groups (F). n = 3 replicates per group, t-test, **P* < 0.05.

**Supplementary Figure 4. Effects of PYY treatment on intestinal mucosal repair following DSS injury.**

(A) Body weight changes of DSS-induced colitis mice treated with PYY (n = 7) or water (n = 6).

(B) Representative images of colon specimens from DSS colitis mice and statistical analysis of colon length.

(C) Histological H&E staining of colon specimens in the DSS model.

(D) Histological scoring of colon specimens in the DSS model. n = 6 mice per group, with 16 fields of view per mice.

(E) qPCR analysis of inflammatory mediators in the colonic mucosa of DSS mice treated with PYY (n = 7) or water (n = 6).

**Supplementary Figure 5. Uncropped blots corresponding to Figure 4E and 5B.**

**Supplementary Table 1. Basic Information and Clinical/Endoscopic Scores at Initial Diagnosis and Follow-up in Crohn's Disease Patients**

| ID | Gender | Age | Initial Diagnosis - Clinical Score | Initial Diagnosis - Endoscopic Score | Follow-up - Clinical Score | Follow-up - Endoscopic Score | Timing of Biopsy for Organoid Culture |
| --- | --- | --- | --- | --- | --- | --- | --- |
| NL01 | Male | 25 | 1 | 25 | 1 | 0 | Follow-up |
| NL02 | Female | 27 | 8 | 12 | 0 | 0 | Follow-up |
| NL03 | Male | 34 | 8 | 18 | 1 | 4 | Follow-up |
| NL04 | Male | 28 | 9 | 27 | NA | NA | Initial Diagnosis |
| NL05 | Male | 22 | NA | NA | 3 | 17 | Follow-up |
| NL06 | Female | 45 | 9 | 22 | 0 | 4 | Follow-up |
| NL07 | Male | 26 | 4 | 17 | 0 | 0 | Follow-up |
| NL08 | Female | 26 | 10 | 10 | 0 | 6 | Follow-up |
| NL09 | Male | 29 | 6 | 23 | 0 | 11 | Follow-up |
| NL10 | Female | 38 | NA | NA | 2 | 15 | Follow-up |
| NL11 | Male | 36 | 8 | 26 | 1 | 17 | Follow-up |
| NL12 | Female | 24 | NA | NA | 0 | 0 | Follow-up |
| NL13 | Female | 22 | 6 | 26 | 3 | 13 | Follow-up |
| NL14 | Male | 35 | 7 | 12 | 0 | 12 | Follow-up |
| NL15 | Female | 27 | 5 | 7 | NA | NA | Initial Diagnosis |
| NL16 | Female | 28 | 3 | 9 | 1 | 0 | Follow-up |
| NL17 | Male | 22 | 5 | NA* | 1 | 23 | Follow-up |
| NL18 | Female | 32 | 2 | 13 | 0 | 10 | Follow-up |
| NL19 | Female | 17 | 5 | NA* | 0 | 22 | Follow-up |

***Note:** The missing endoscopic scores for this individual were due to the endoscopic examination being performed at another hospital.

**Supplementary Table 2 Primers Used for qPCR Detection in This Study**

| **Gene** | **Species** | **Type** | **Sequence** |
| --- | --- | --- | --- |
| Gapdh | Mouse | F | 5’-AGGTCGGTGTGAACGGATTTG-3’ |
|  |  | R | 5’-GGGGTCGTTGATGGCAACA-3’ |
| Hes1 | Mouse | F | 5’-TCAGCGAGTGCATGAACGAG-3’ |
|  |  | R | 5’-CATGGCGTTGATCTGGGTCA-3’ |
| Atoh | Mouse | F | 5’-GAGTGGGCTGAGGTAAAAGAGT-3’ |
|  |  | R | 5’-GGTCGGTGCTATCCAGGAG-3’ |
| Spdef | Mouse | F | 5’-AAGGCAGCATCAGGAGCAATG-3’ |
|  |  | R | 5’-CTGTCAATGACGGGACACTG-3’ |
| Neurog3 | Mouse | F | 5’-AGTGCTCAGTTCCAATTCCAC-3’ |
|  |  | R | 5’-CGGCTTCTTCGCTTTTTGCTG-3’ |
| Chga | Mouse | F | 5’-ATCCTCTCTATCCTGCGACAC-3’ |
|  |  | R | 5’-GGGCTCTGGTTCTCAAACACT-3’ |
| Pparg | Mouse | F | 5’-TCGCTGATGCACTGCCTATG-3’ |
|  |  | R | 5’-GAGAGGTCCACAGAGCTGATT-3’ |
| PPARG | Human | F | 5’-ACCAAAGTGCAATCAAAGTGGA-3’ |
|  |  | R | 5’-ATGAGGGAGTTGGAAGGCTCT-3’ |
| ADRP | Human | F | 5’-TTGCAGTTGCCAATACCTATGC-3’ |
|  |  | R | 5’-CCAGTCACAGTAGTCGTCACA-3’ |
| CD36 | Human | F | 5’-AAGCCAGGTATTGCAGTTCTTT-3’ |
|  |  | R | 5’-GCATTTGCTGATGTCTAGCACA-3’ |
| FABP | Human | F | 5’-AAGACAGTGGTTCAGTTGGAAG-3’ |
|  |  | R | 5’-TGAGTTCGGTCACAGACTTGAT-3’ |
| LPL | Human | F | 5’-AGGATGTGGCCCGGTTTATC-3’ |
|  |  | R | 5’-CCAAGGCTGTATCCCAAGAGAT-3’ |

**Supplementary Methods**

**Histology, immunohistochemistry (IHC), and immunofluorescence (IF)**

Hematoxylin and eosin (HE) staining was performed to standard protocols, as previously described. Histological damage scoring was also conducted according to previously published protocols ^1^. Scoring for crypt damage, depth of inflammation, and total inflammation was similar to our previous study ^2^. All histology was assessed by QuPath ^3^ and validated by qualified pathologists in a blinded manner. IHC or IF was performed as previously described ^2^. Antibodies included Satb2 (Santa Cruz, sc-81376; Abclonal, A20220), Muc2 (Abcam, ab272692), ChgA (Santa Cruz, sc-376827), Dclk1 (Abcam, ab109029), GLP-1 (NOVUS, NBP2-23558), PYY (Cell Signaling Technology, 24895), and Ki67 (Cell Signaling Technology, 12202).

**Flow cytometry**

The colon was opened longitudinally, rinsed with PBS, and cut into 2-cm fragments. These fragments were transferred to a 15-ml conical tube (Falcon 2070) with 10 ml EDTA-DTT-PBS solution containing 30 mM EDTA and 1.5 mM DTT. The tube was incubated in a water bath at 37℃ for 10 minutes and then shaken at an acceleration of 2.5g-3.5g along the long axis for 30 seconds. The residual intestinal tissue was discarded, and the crypts were collected by centrifuging at 1000g at 4℃ for 5 minutes. After discarding the supernatant, the crypts were re-suspended in DPBS containing 10% FBS and centrifuged again at 1000g at 4℃ for 5 minutes. The supernatant was discarded, and the crypts were transferred to a 15ml conical tube containing 10 ml of Dispase-HBSS solution, including 0.3 U/ml Dispase. The tube was placed in a water bath at 37℃ and shaken at an acceleration of 3-3.5 g along the long axis for 15 seconds every 2 minutes for a total of 4 cycles to separate the crypts into single cells. Subsequently, the cells were sequentially filtered through a 70-μm and a 40-μm nylon mesh to remove cell debris and sticky cells, centrifuged at 1000 g at 4℃ for 5 minutes, re-suspended in HBSS for washing, and then re-suspended in 1ml of DMEN medium for cell counting. Aliquots of 1 × 10^6^ cells of the above tissues were incubated with antibodies or solutions, including Zombie Aqua solution (Biolegend, 423101), EpCAM antibody (Biolegend, 118206), fixation / permeabilization solution (Thermo Fisher Scientific, 00-5523-00), Ki67 (Biolegend, 652411), and Satb2 (Abcam, ab196536). Flow cytometry was performed using FACScan (BD Biosciences), and data were analyzed by FlowJo software (version X.0.7; Tree Star Inc.).

**Organoid culture and treatment**

Murine colons were longitudinally opened, cut into small pieces, and washed with cold Dulbecco phosphate-buffered saline (DPBS) using a 10-mL pipette pre-wetted with DPBS containing 1% BSA. The tissue pieces were allowed to settle for 30 seconds to facilitate sedimentation through gravitational forces. The resuspension/sedimentation procedure was repeated typically 15–20 times until the supernatant became clear. Subsequently, the tissue pieces were incubated in 2-5 mmol/L EDTA cold buffer for 20 minutes on ice. After removing the EDTA buffer, the tissue pieces were resuspended in cold DPBS containing 0.1% BSA. Using a 10-mL pipette to isolate intestinal crypts, they were settled for 30 seconds to facilitate sedimentation through gravitational forces, and the supernatant was removed. This resuspension/sedimentation procedure was typically performed 6–8 times. The supernatant was then filtered through a 70-μm nylon mesh into a clean 50ml tube to obtain the first fraction. This step was repeated 4 times until the supernatant did not contain crypts. The mixture was then centrifuged at 200g for 3 minutes to separate crypts from single cells. The precipitate was resuspended with an appropriate amount of DMEM/F12 medium and Matrigel, and 30 μl of the mixture was aspirated and added to the center of each well of a preheated 48-well plate to form a dome. After incubating it for 10 minutes, 200 μl of complete organoid growth medium at room temperature was added to each well.

A 48-well plate containing colonic crypt-like organoids was exposed to X-ray irradiation within a biological irradiator. Specific parameters were set as follows: a source-plate distance of 40 cm, an absorbed dose rate of 1.225 Gy/min, and a radiation duration of 98 s. The cumulative radiation dose reached 2 Gy. Subsequently, organoid cultures derived from Satb2 knockout mice and control mice were photographed. A comparative analysis was conducted to assess the morphology, survival rate of the organoids, and the number of buds per surviving organoid.

Fresh intestinal biopsy specimens were placed in tissue preservation solution (Mogengel Biotech, MA-0817H001S) and transported to the laboratory on the same day for organoid culture. The method for isolating intestinal crypts was as previously described, and the organoid culture method was also the same, with the exception that the organoid growth medium (Mogengel, MA-0817H001S) was different. Organoids were passaged after approximately 3 weeks of culture. One day after passaging, PYY was added at a concentration of 10 pmol for 4 consecutive days. Organoid survival rates and budding numbers were recorded, and on day 10, organoid cultures were collected for quantitative PCR to assess the expression differences of PPAR-γ downstream genes. The experimental methods were the same as previously described.

**Cell culture**

The human colorectal cancer cell lines, including HCT116, RKO, and CACO2, as well as the 293T cell line, were obtained from the Cell Bank of the Chinese Academy of Sciences (Shanghai, China). The cell culture procedures and authentication methods were previously described ^2^.

**RNA extraction, real-time polymerase chain reaction**

Mice mucosal tissue RNA extraction was similar to our previous study. Real-time polymerase chain reaction (RT-PCR) was conducted using SYBR Green Mix (Applied Biosystems, US); primers are listed in ***Supplementary Table 2***.

**Nuclear Extraction**

To examine the intracellular localization of PYY, Caco2 and 293T cell lines were treated with PYY (10 nmol) in their respective culture media. After incubation periods of 4, 8, 12, and 24 hours, cytoplasmic and nuclear proteins were extracted using the Beyotime Kit (Cat. No.: P0028). Subsequently, immunoblotting techniques were employed to assess the presence of PYY in both cytoplasmic and nuclear protein extracts, following established protocols^2^.

**Luciferase assay and ATP measurement**

The siRNAs for SATB2 and negative control (NC) were the same as our previous study ^1^. Transient transfection was conducted using Lipofectamine 2000 (Invitrogen, USA) according to the manufacturer’s protocol. Cells were lysed 48 hours post-transfection, and the fluorescence intensity was measured using a luciferase reporter assay system (Promega). Each transfection included β-gal as a control. Experiments were performed in triplicate wells and repeated at least three times. In Caco2 and 293T cells, 100 pmol of PYY or a scrambled peptide control were added, and ATP content was measured using the ATP Chemiluminescence Assay Kit (Elabscience, E-BC-F002).

**Reference**

1. Ni, H., Chen, Y., Xia, W., Wang, C. Y., Hu, C., Sun, L., *et al.* SATB2 Defect Promotes Colitis and Colitis-associated Colorectal Cancer by Impairing Cl-/HCO3- Exchange and Homeostasis of Gut Microbiota. *J Crohns Colitis* **15**, 2088-2102 (2021). https://doi.org:10.1093/ecco-jcc/jjab094

2. Ni, H., Chen, L., Song, L., Sun, L., Cui, H., Zhou, Q.*, et al.* ErbB4 acts as a suppressor in colitis and its associated carcinoma by negatively regulating cholesterol metabolism. *Carcinogenesis* **40**, 680-686 (2019). https://doi.org:10.1093/carcin/bgy164

3. Bankhead, P., Loughrey, M. B.*,* Fernández, J. A., Dombrowski, Y., McArt, D. G., Dunne P. D., *et al.* Qupath: Open source software for digital pathology image analysis. *Sci Rep* 2017;**7**:16878.
