# Supplementary figures and images for: The protective role of PYY in intestinal mucosal defects induced by SATB2 deficiency in inflammatory bowel disease
Source: Cell Death Discov. 2025 May 9;11:227. doi: 10.1038/s41420-025-02511-y (PMC12062304; doi:10.1038/s41420-025-02511-y)

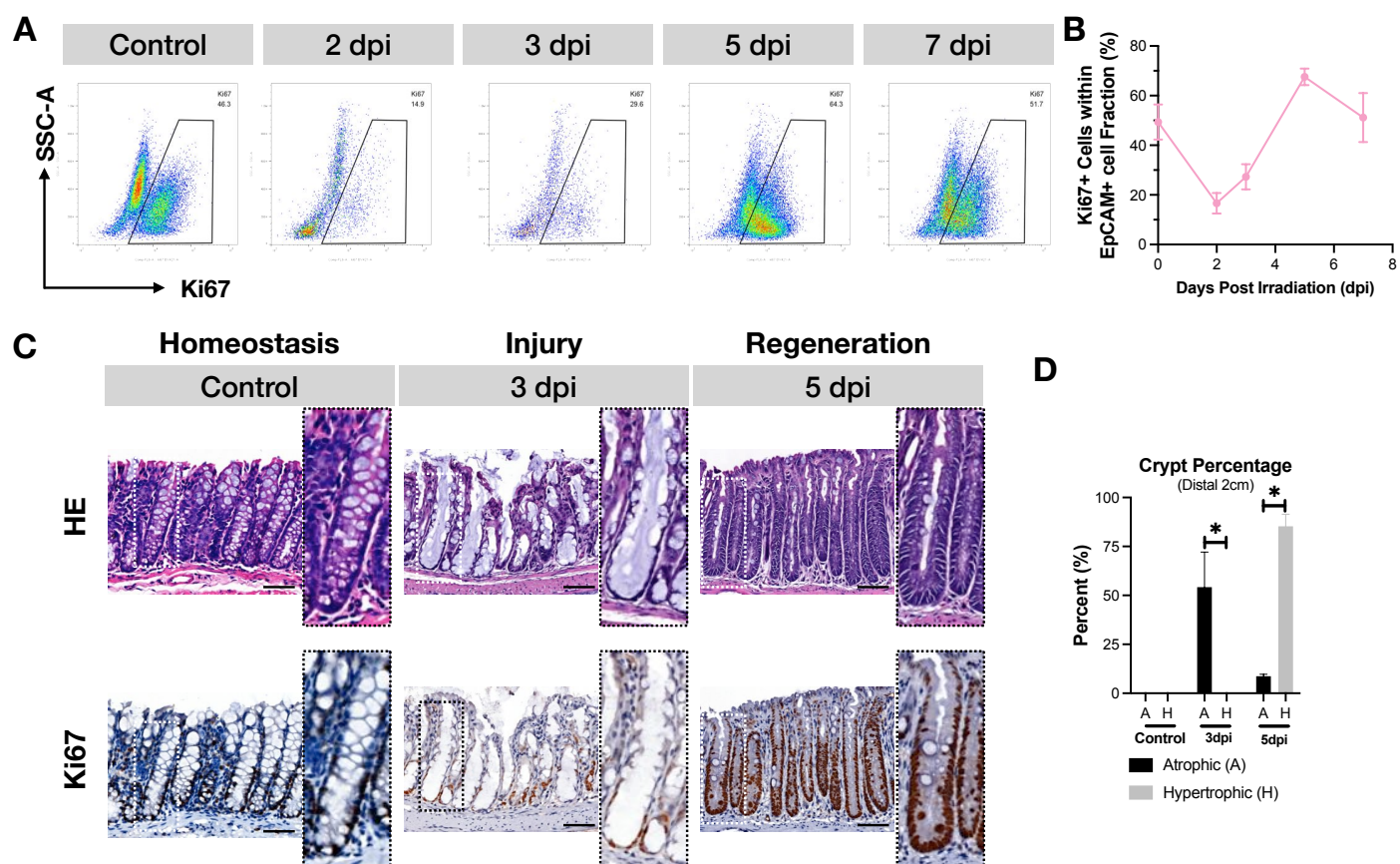

Supplementary Figure 1

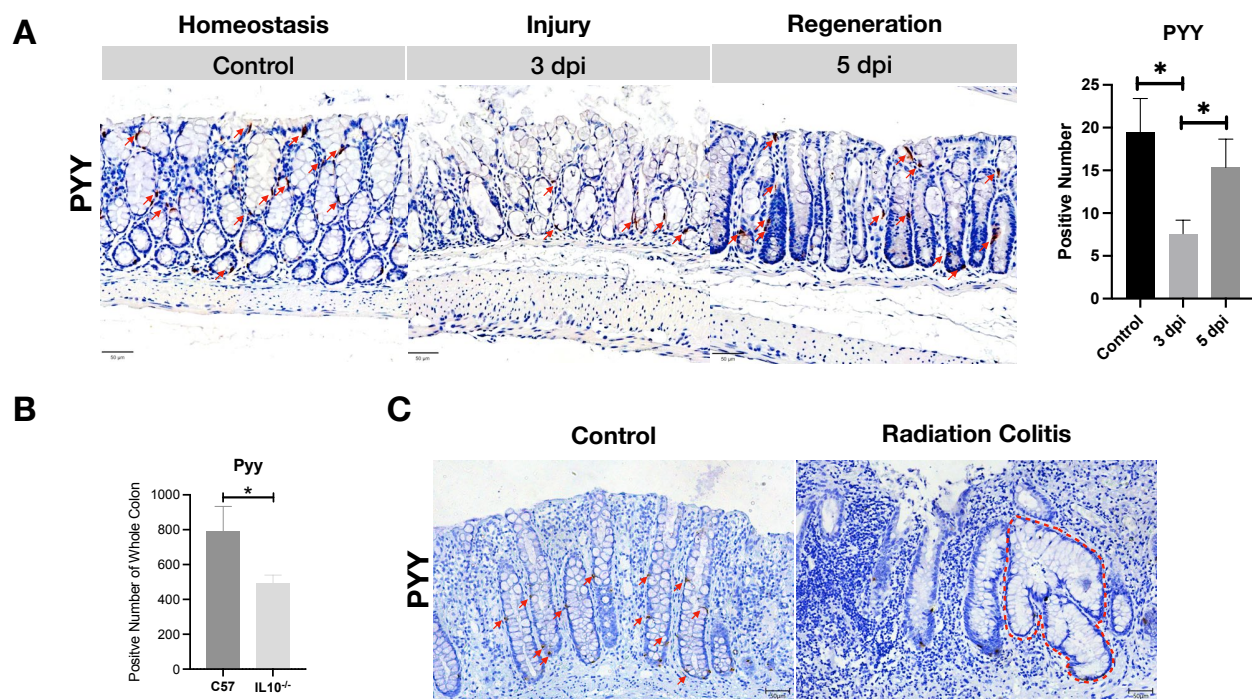

**Supplementary Figure 2**

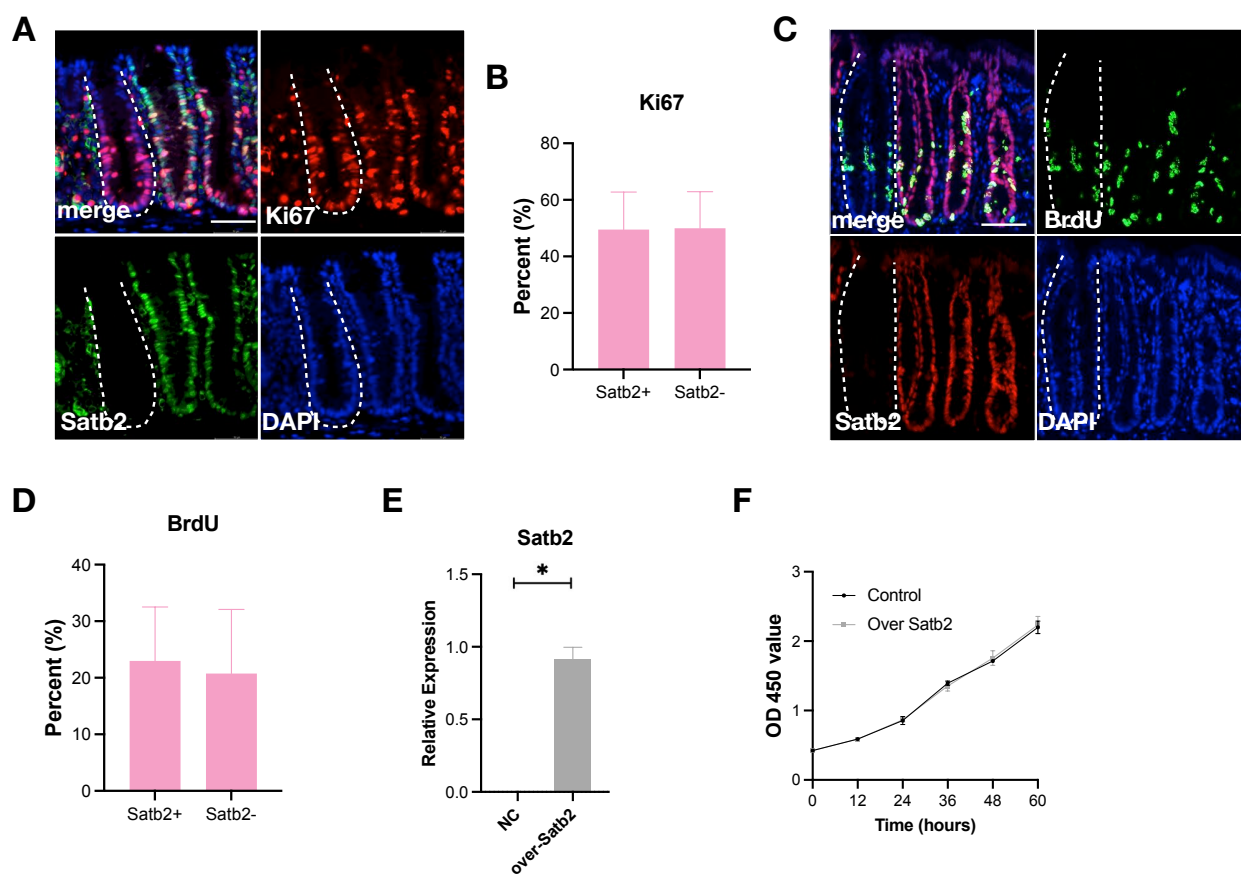

**Supplementary Figure 3**

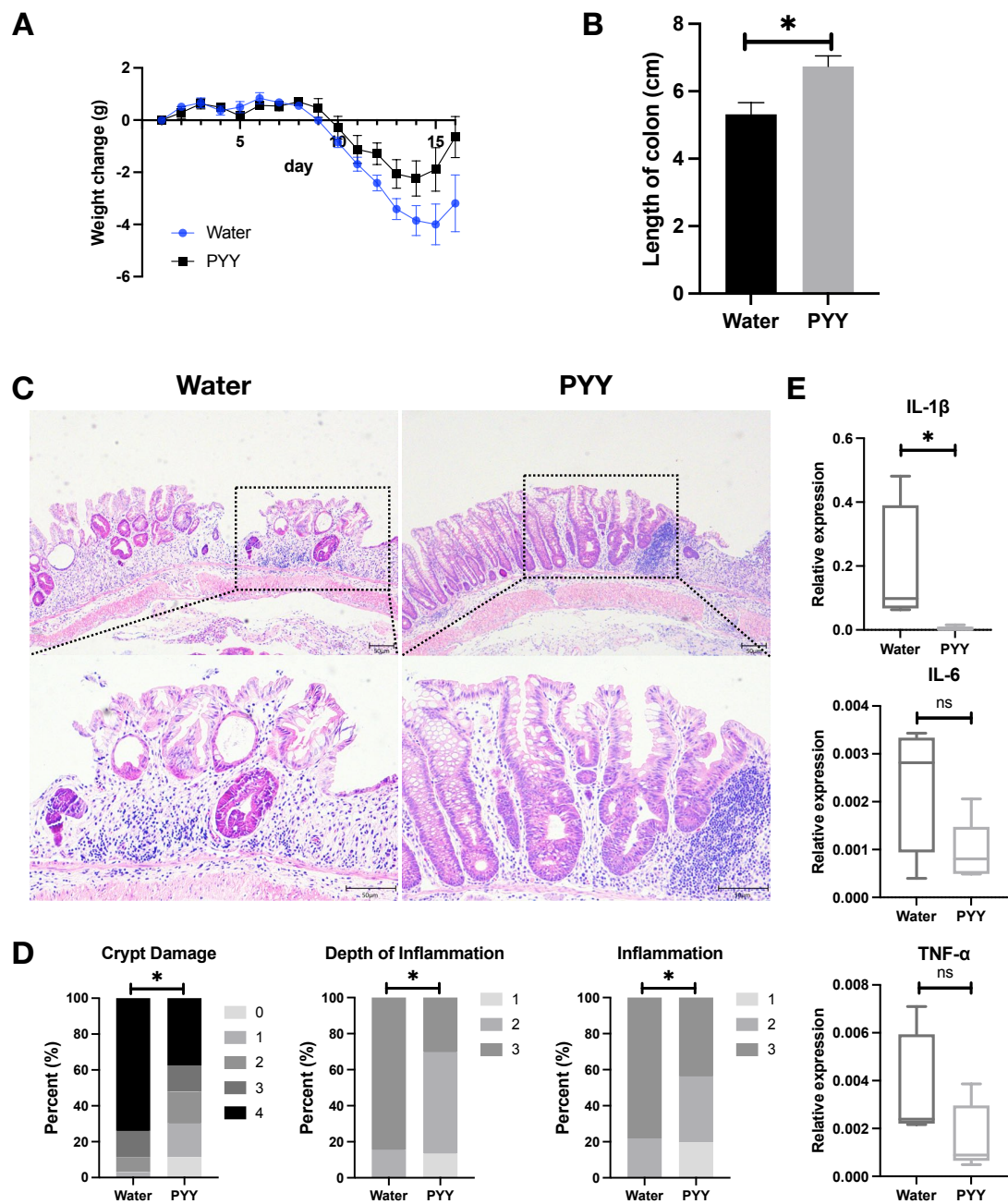

**Supplementary Figure 4**

Supplement: Supplementary file 2 — Supplementary Figures [file 41420_2025_2511_MOESM2_ESM.pdf]
