# Supplementary material for: The protective role of PYY in intestinal mucosal defects induced by SATB2 deficiency in inflammatory bowel disease
Source: Cell Death Discov. 2025 May 9;11:227. doi: 10.1038/s41420-025-02511-y (PMC12062304; doi:10.1038/s41420-025-02511-y)

Full length uncropped original western blots corresponding to Figure 4E.

RKO
PPAR-γ

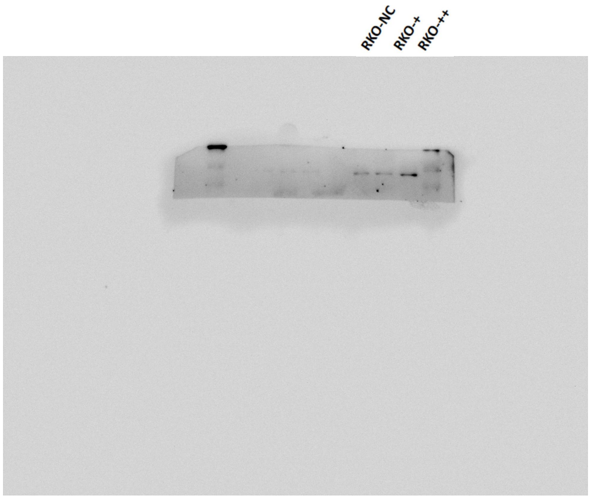

HCT116
PPAR-γ

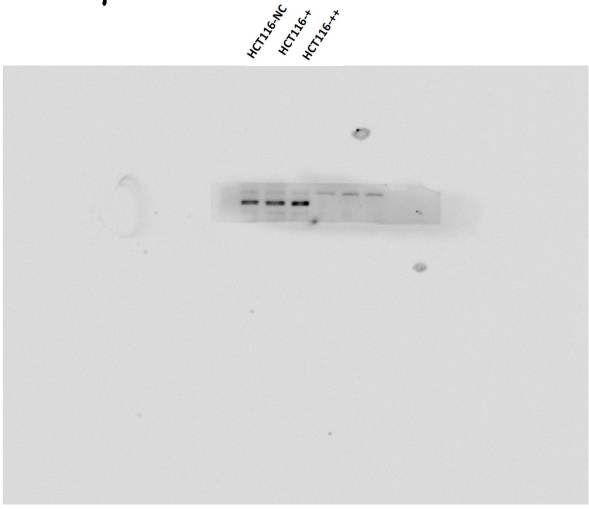

RKO
GAPDH

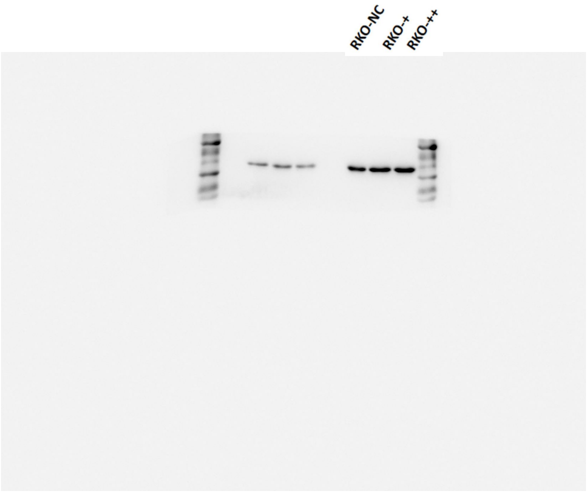

HCT116
GAPDH

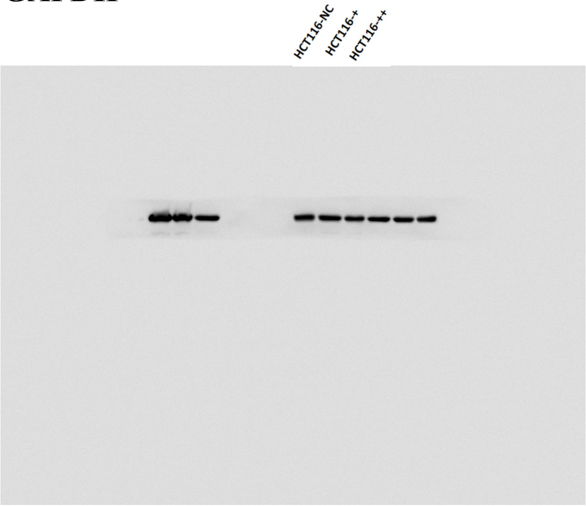

Full length uncropped original western blots corresponding to Figure 5B.

Caco2
Cytoplasm-PYY

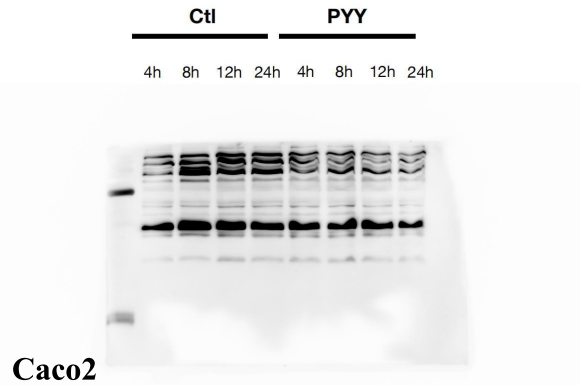

Caco2
Nucleus-PYY

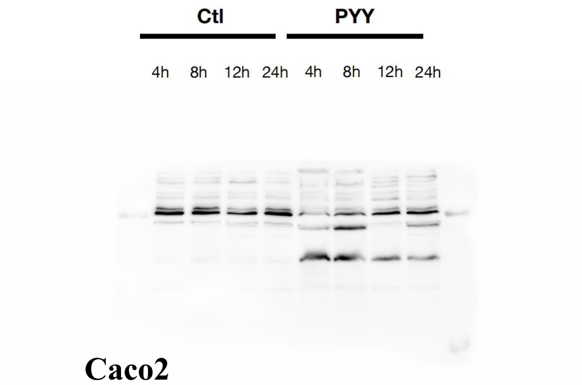

Caco2
Cytoplasm-GAPDH

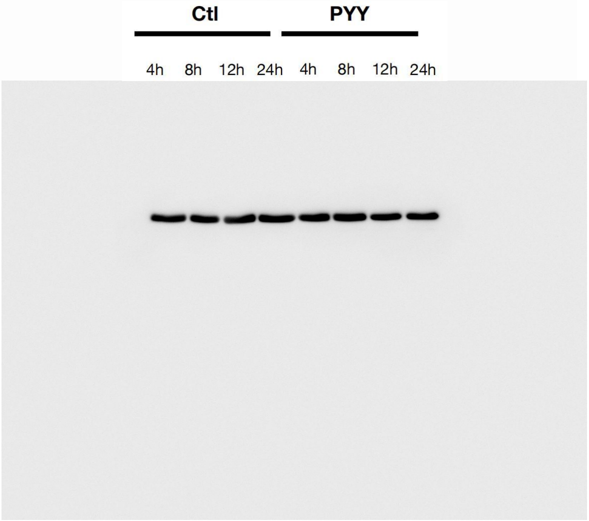

Caco2
Nucleus-histone H3

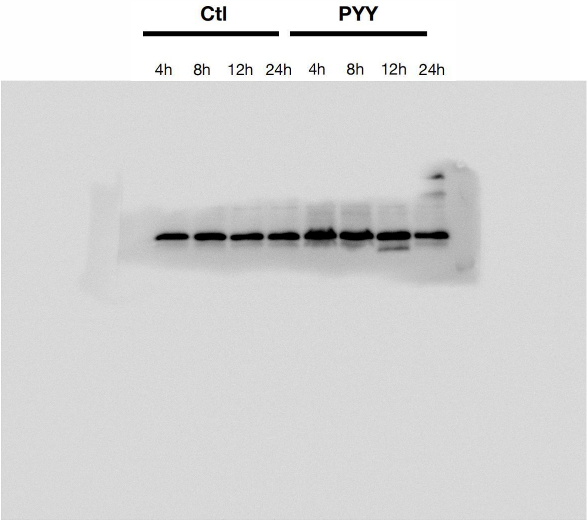

293T
Cytoplasm-PYY

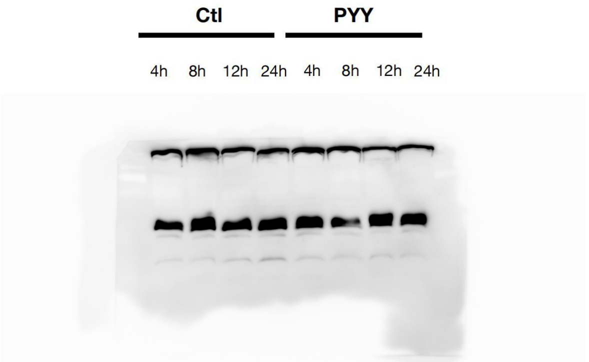

293T
Nucleus-PYY

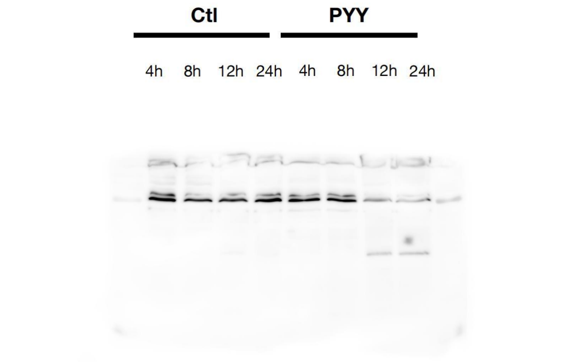

293T
Cytoplasm-GAPDH

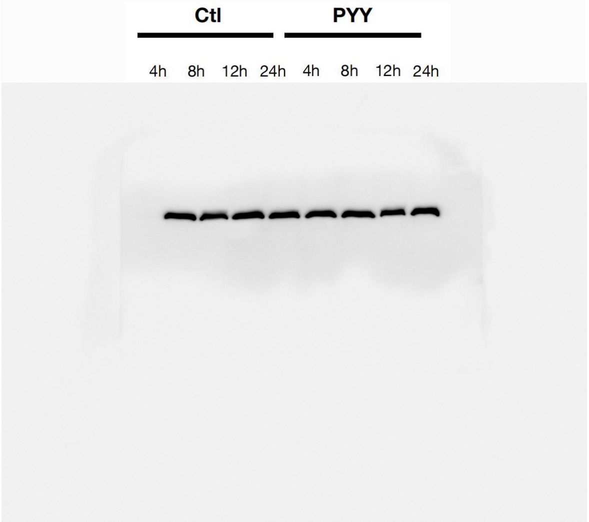

293T
Nucleus-histone H3

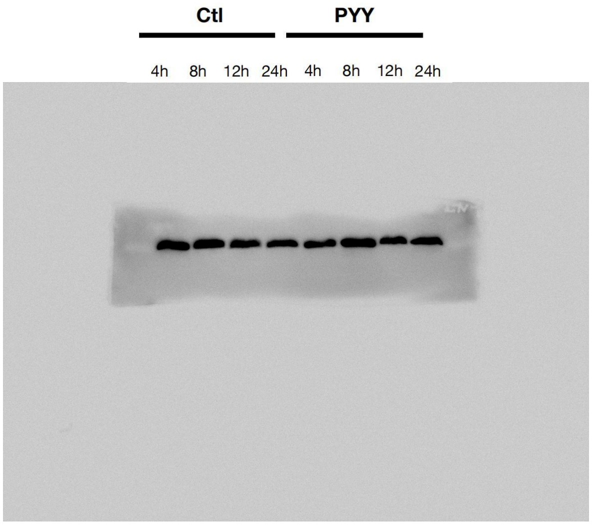

Supplement: Supplementary file 3 — Original Data [file 41420_2025_2511_MOESM3_ESM.pdf]
